# Molecular Prevalence and *gp60* Subtype Diversity of *Cryptosporidium* spp. in Dairy Cattle in Anhui, China

**DOI:** 10.3390/ani16162467

**Published:** 2026-08-08

**Authors:** Falei Li, Hui Liu, Yingying Zhao, Zhenyi Zi, Xiaohui Wang, En Liu, Jinbo Hou, Huilin Zhang

**Affiliations:** 1 Anhui Province Key Laboratory of Embryo Development and Reproductive Regulation, Anhui Province Key Laboratory of Pollution Damage and Biological Control for Huaihe River Basin, College of Biological and Food Engineering, Fuyang Normal University, Fuyang 236037, China; fli@fynu.edu.cn (F.L.);; 2 Fuyang City Animal Disease Prevention and Control Center, Fuyang 236037, China

**Keywords:** *Cryptospridium* spp., prevalence, dairy cattle, zoonosis, China

## Abstract

*Cryptosporidium* spp. are important gastrointestinal disease-causing parasites found in humans and animals. Cattle are major hosts for *Cryptosporidium* species such as *C. parvum*, *C. bovis*, *C. ryanae*, and *C. andersoni*, with *C. parvum* being the most pathogenic and a key zoonotic agent. However, systematic epidemiological data on *Cryptosporidium* in dairy cattle from Anhui Province remain scarce. In this study, we aim to investigate its prevalence and genetic diversity. *Cryptosporidium* species and subtypes were identified by targeting the small subunit ribosomal RNA (*SSU* rRNA) and 60 kDa glycoprotein (*gp60*) genes. PCR testing revealed an overall *Cryptosporidium* infection rate of 35.7% (338/948) across four dairy farms in Anhui Province, with significant regional variation (*p* < 0.01). Bengbu exhibited the highest infection rate (61.5%), whereas the lowest was found in Fuyang (8.6%). Age-specific distribution indicated that calves aged 2–6 months exhibited the highest infection rate (50.2%). Four *Cryptosporidium* spp. were identified, *C. parvum*, *C. andersoni*, *C. ryanae*, and *C. bovis*, with *C. parvum* and *C. bovis* being predominant. All *C. parvum* subtyped isolates belonged to the IId subtype family. Seven and five *gp60* subtype families were detected in *C. ryanae* and *C. bovis*, respectively. Our findings revealed distinct geographical and age-associated variations in the distribution of *Cryptosporidium* spp. in dairy cattle in Anhui Province, highlighting the need for targeted control strategies in high-risk regions and young calves.

## Simple Summary

*Cryptosporidium* spp. are common intestinal pathogens found in humans and other farmed animals, particularly cattle. Limited data are available on the positivity rates and genetic characteristics of *Cryptosporidium* spp. in dairy cattle in Anhui, China. In this study, a total of 948 faecal samples were analysed via nested PCR targeting the small subunit ribosomal RNA (*SSU* rRNA). The identified *C. parvum*, *C. ryanae*, and *C. bovis* samples were analysed by targeting 60 kDa glycoprotein (*gp60*) genes. PCR testing revealed an overall *Cryptosporidium* infection rate of 35.7% (338/948) across four dairy farms in Anhui Province. Four *Cryptosporidium* spp. were identified: *C. parvum*, *C. andersoni*, *C. ryanae*, and *C. bovis*. The IId subtype family dominated *C. parvum* isolates. Seven *gp60* subtype families were detected in *C. ryanae*, and five distinct subtype families were identified in *C. bovis*. The data obtained suggest that the zoonotic *C. parvum* subtypes are commonly found in these animals, providing support for the development of effective prevention and control strategies against intestinal infections in cattle in Anhui, China.

## 1. Introduction

*Cryptosporidium* spp. are classified within Apicomplexa, Sporozoa, Gregarinomorphea, Cryptogregaria, Cryptogregarida, Cryptosporidiidae, and *Cryptosporidium* [1]. *Cryptosporidium* can cause acute gastroenteritis, abdominal pain, and diarrhoea in a variety of vertebrates, including humans [2]. Research indicates that *Cryptosporidium* spp. were a leading cause of moderate-to-severe gastrointestinal illness in children under 5 years old and cattle under 2 months [3].

To date, 47 *Cryptosporidium* species and more than 120 genotypes have been identified in humans and animals, with most being host-adapted [4]. Dairy cattle can be infected with a wide variety of *Cryptosporidium* species, the most common being *C. parvum*, *C. andersoni*, *C. ryanae* and *C. bovis*. In addition, other species such as *C. felis*, *C. ubiquitum*, *C. serpentis*, *C. meleagridis*, *C. xiaoi*, *C. hominis*, *C. muris*, and *C. suis* have been found in dairy cattle [4,5,6,7]. *Cryptosporidium* spp. occurrence in dairy cattle is strongly associated with host age [8]. *Cryptosporidium andersoni* infects the abomasum of adult cattle, affecting milk production [9]. *Cryptosporidium bovis* and *C. ryanae* primarily infect post-weaning calves, which typically exhibit no clinical signs of disease [10,11]. In contrast, *C. parvum* predominantly infects pre-weaned calves [12]. Although *C. bovis*, *C. ryanae*, and *C. andersoni* have been detected in calves before weaning, infection in pre-weaned calves is primarily caused by *C. parvum* and often leads to neonatal diarrhoea [8]. *Cryptosporidium parvum* is recognised as the leading pathogen of zoonotic infections between humans and animals, and is also one of the principal causes of neonatal enteritis in ruminants worldwide [13,14]. As potential reservoirs of human cryptosporidiosis, dairy cattle can transmit the parasite both directly through contact and indirectly via manure-mediated contamination of soil and water, thereby increasing the risk of infection among humans and other susceptible animals and contributing to the environmental contamination.

The typical subtyping method for *Cryptosporidium* spp. is based on sequence analysis of the 60 kDa glycoprotein (*gp60*) gene [15]. Among the four most common *Cryptosporidium* species identified in dairy cattle, *gp60*-based subtyping tools have been developed for *C. parvum*, *C. ryanae*, and *C. bovis* but remain unavailable for *C. andersoni*. [16,17,18]. For *C. parvum*, more than 20 subtype families have been identified [19]. Among them, IIa, IId, and IIl subtype families have been identified in cattle [4]. Notably, the distribution of these subtype families exhibits marked geographic variation. In European countries and New Zealand, cattle cryptosporidiosis due to *C. parvum* is predominantly attributed to the IIa subtype family [2]. In certain countries, including China, the IId subtype family represents the only *C. parvum* subtype family in cattle, whereas the IIl subtype family has only been documented in Europe [20,21]. For *C. bovis*, six subtype families have been described, ranging from XXVIa to XXVIf, and no marked host adaptation or geographic variation has been observed [18]. For *C. ryanae*, eleven subtype families have been identified, ranging from XXIa to XXIk, and geographical variation in the distribution of subtype families has been observed in dairy cattle [17].

The rapid expansion of the dairy cattle industry in Anhui Province in recent years has heightened the risk of soil and water contamination with oocysts, in addition to increasing occupational exposure of dairy farm personnel to *Cryptosporidium* spp., thereby facilitating potential zoonotic transmission of this pathogen. Despite these potential risks, only one study has been performed to date to determine the identity of *Cryptosporidium* spp. in dairy cattle in this region [22]. To elucidate the transmission and zoonotic potential of *Cryptosporidium* spp. in dairy cattle, we determined the prevalence and conducted genetic characterisation of this pathogen in dairy cattle in Anhui Province, China.

## 2. Materials and Methods

### 2.1. Ethical Approval

Faecal samples from cattle on four farms in Anhui Province, China, were collected with the consent of the farmers in line with established practices. The research protocol was approved by the Research Ethics Committee of the Fuyang Normal University (No. FYNU2023AP024).

### 2.2. Specimens

**Figure 1:**
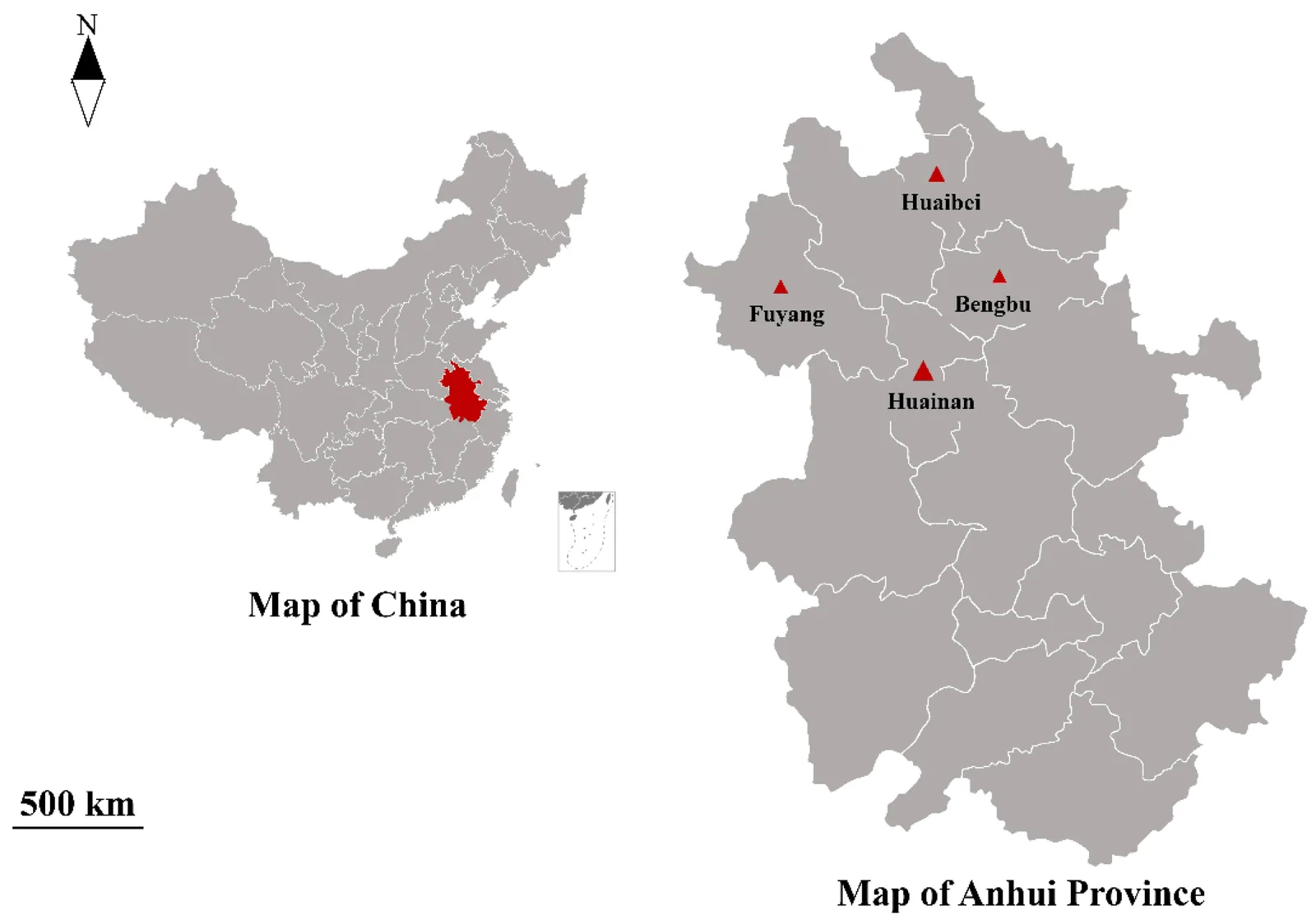
Map of China indicating the regions where specimens used in this study were collected.

A total of 948 faecal samples were collected from female cattle on four farms (Fuyang, Huainan, Huaibei and Bengbu) in Anhui Province, China, between May 2023 and August 2024 (Figure 1). During sample collection, several pre-weaned calves were observed to have diarrhoea. The farms in Bengbu, Huaibei, Huainan, and Fuyang were established in 2011, 2018, 2021, and 2023, respectively. Among the faecal samples, 234, 167, 238, and 309 were collected from Fuyang, Huainan, Huaibei, and Bengbu, respectively. All faecal samples were obtained from intensive dairy farming systems. All cattle were segregated by age, with calves under two months specifically housed apart from older animals. Based on age, the cattle were stratified into four groups: <2 months (n = 323), 2–6 months (n = 359), 6–12 months (n = 155), and >12 months (n = 111). After donning gloves, fingers were gently inserted into the animal’s rectum. Once the animal exhibited a defecation response, the freshly excreted faecal samples were collected. The samples were placed in a sealed plastic bag, and the age of the cattle and the time and place of sample collection were recorded. All samples were stored in 2.5% potassium dichromate at 4 °C prior to use for DNA extraction.

### 2.3. DNA Extraction

Approximately 200 mg of each bovine faecal sample was washed three times with distilled water via centrifugation. Genomic DNA was then extracted using an E.Z.N.A.^®^ Stool DNA Kit (Omega Bio-Tek, Inc., Norcross, GA, USA) following the manufacturer’s instructions. The purified DNA was stored at −20 °C until being used for *Cryptosporidium* species identification and subtype determination.

### 2.4. Detection of Cryptosporidium spp.

To detect *Cryptosporidium* spp., a nested PCR targeting an 830 bp fragment of the *SSU* rRNA gene was performed on all samples [23]. For the identified positive samples of *Cryptosporidium* species, PCR amplification was performed using the *gp60* gene to identify *Cryptosporidium* subtypes [17,18,24]. Positive and negative samples were included in each PCR assay. To ensure reliability, PCR was performed in duplicate for each sample at each locus. A sample was considered positive if either of the two independent reactions yielded the expected amplicon of the correct size.

### 2.5. Sequence Analysis

All positive products of the second PCR were sequenced bi-directionally on an ABI 3730 Autosequencer (Applied Biosystems, Foster City, CA, USA) to identify the *Cryptosporidium* spp. presented. The nucleotide sequences were assembled using ChromasPro 2.1.5.0 (http://technelysium.com.au/ChromasPro.html, accessed on 10 May 2026), edited using BioEdit 7.1.3.0 (http://thalljiscience.github.io, accessed on 18 May 2026), and aligned using Clustal2 (http://www.clustal.org/clustal2/, accessed on 28 May 2026). Phylogenetic analysis of sequences generated in this study was performed by constructing maximum likelihood trees using Mega 7.0 (http://www.megasoſtware.net/, accessed on 10 May 2026) under the general time reversible model, with 1000 bootstrap replicates to evaluate node support.

### 2.6. Statistical Analysis

The Chi-square test implemented in SPSS 20.0 (IBM Inc., Chicago, IL, USA) was used to compare positive rates of *Cryptosporidium* spp. between geographical areas and age groups. Differences with *p* < 0.05 were considered to be significant.

## 3. Results

### 3.1. Occurrence of Cryptosporidium Species in Dairy Cattle

PCR testing revealed a *Cryptosporidium* prevalence of 35.7% (338/948) across four dairy farms in Fuyang, Huainan, Huaibei, and Bengbu (Table 1). The positivity rates of *Cryptosporidium* spp. were 8.5% (20/234), 19.8% (33/167), 39.9% (95/238), and 61.5% (190/309) in Fuyang, Huainan, Huaibei, and Bengbu, respectively (Table 1). Based on the chi-square test results, the differences in *Cryptosporidium* infection among dairy cattle in different cities exhibited significant spatial heterogeneity (*p* < 0.0001). Among them, the positivity rate of *Cryptosporidium* from dairy cattle in Bengbu was significantly higher than that in Fuyang (*χ*^2^ = 157.4, *df* = 1, *p* < 0.0001), Huainan (*χ*^2^ = 82.5, *df* = 1, *p* < 0.0001), and Huaibei (*χ*^2^ = 25.1, *df* = 1, *p* < 0.0001). Similarly, the detection rates from dairy cattle in Huaibei were significantly higher than those in Huainan (*χ*^2^ = 18.4, *df* = 1, *p* < 0.0001) and Fuyang (*χ*^2^ = 63.0, *df* = 1, *p* < 0.0001).

Across age groups, the positivity rates of *Cryptosporidium* in dairy cattle under 2 months, 2–6 months, 6–12 months, and >12 months were 27.2% (88/323), 43.1% (155/359), 30.9% (48/155), and 42.3% (47/111), respectively (Table 2). The positivity rates of the 2–6-month group and >12-month group were significantly higher than that of the group under 2 months (*χ*^2^ = 18.8, *df* = 1, *p* < 0.0001; *χ*^2^ = 8.8, *df* = 1, *p* = 0.0030, respectively). No significant differences were observed in *Cryptosporidium* detection rates between 2–6-month animals and >12-month animals.

### 3.2. Distribution of Cryptosporidium Species in Dairy Cattle

The secondary PCR products from all 338 *Cryptosporidium*-positive samples were successfully sequenced, revealing four known *Cryptosporidium* species: *C. bovis* (n = 125), *C. parvum* (n = 124), *C. ryanae* (n = 65), and *C. andersoni* (n = 24). By farm, *C. parvum* was the dominant *Cryptosporidium* species in animals in Huainan (23/33) and Huaibei (55/95). In contrast, *C. bovis* was the dominant *Cryptosporidium* species in animals in Fuyang (18/20) and Bengbu (83/190). Furthermore, *C. parvum* was absent in dairy cattle in Fuyang. The nucleotide sequences obtained in this study from *C. bovis*, *C. parvum*, *C. ryanae*, and *C. andersoni* were identical to GenBank sequences MT950118, MK252646, JN400880, and JN400881, respectively.

### 3.3. Subtypes of C. parvum, C. bovis, and C. ryanae

**Figure 2:**
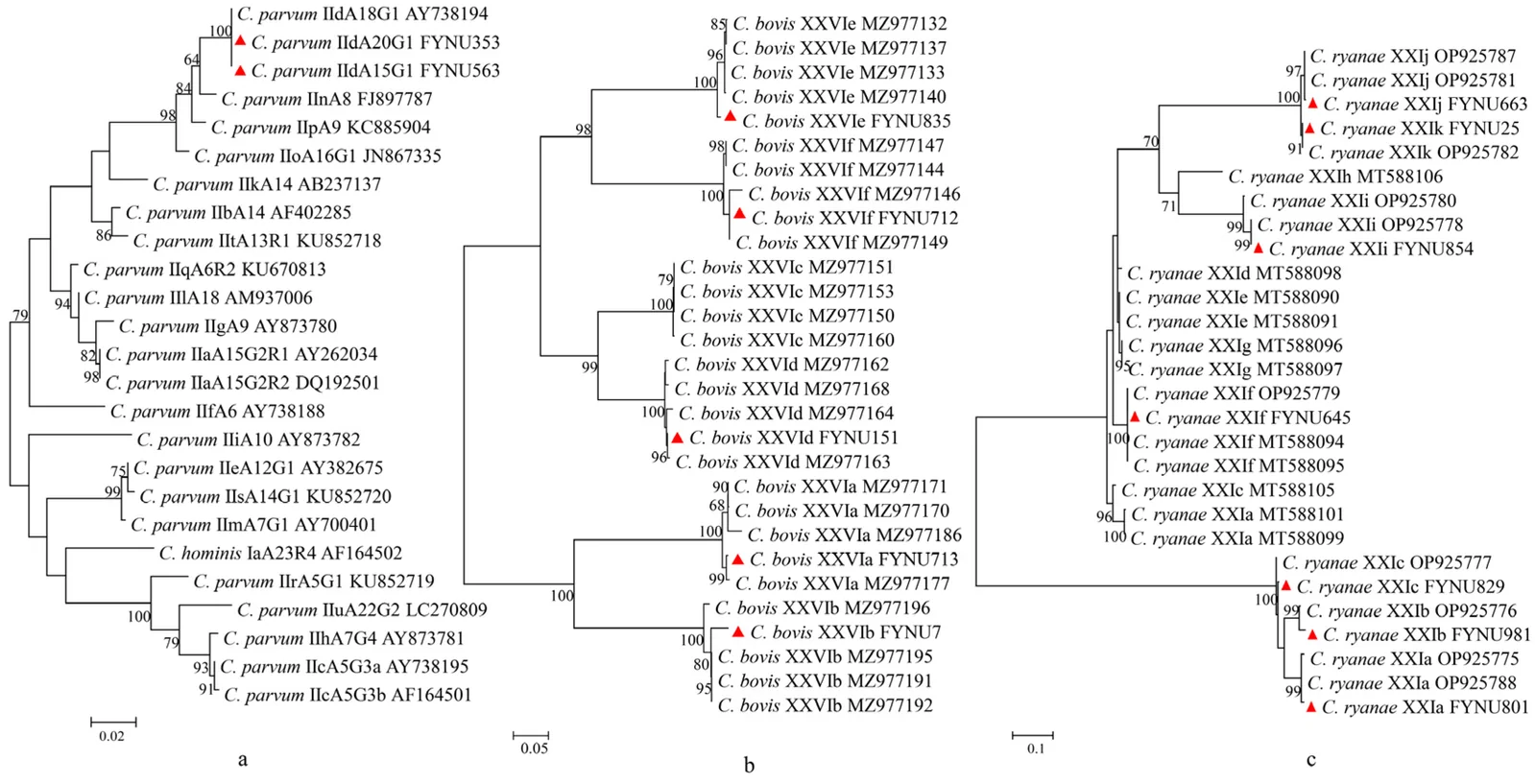
Phylogenetic relationship of *C. parvum* (**a**), *C. bovis* (**b**), and *C. ryanae* (**c**) based on the maximum likelihood analyses of the *gp60* gene (alignment lengths: 735 bp for *C. parvum*, 1388 bp for *C. bovis*, and 1253 bp for *C. ryanae*). Bootstrap values greater than 50% from 1000 replicates are displayed. The red triangles indicate the *Cryptosporidium* subtypes identified in this study.

Among the 124 *C. parvum*-positive specimens, 98 were successfully subtyped via *gp60* gene sequence analysis. Among them, subtypes IIdA15G1 (n = 34) and IIdA20G1 (n = 64) were identified in dairy cattle. The subtype IIdA20G1 was detected in dairy cattle from Huainan and Huaibei, whereas the subtype IIdA15G1 occurred only in dairy cattle from Bengbu (Table 1). The nucleotide sequences of IIdA15G1 and IIdA20G1 were identical to the GenBank reference sequences MW055930 and KT235714, respectively. In the maximum likelihood analysis of the obtained sequences, IIdA15G1 and IIdA20G1 clustered within the *C. parvum* IId subtype family (Figure 2a). Of the 125 *C. bovis*-positive samples, 99 were successfully assigned to five subtype families: XXVIa (n = 5), XXVIb (n = 9), XXVId (n = 9), XXVIe (n = 39), and XXVIf (n = 37). The nucleotide sequences of XXVIa and XXVIf were identical to the GenBank reference sequences MZ977177 and MZ977149, respectively. The sequences from XXVId contained five single-nucleotide polymorphisms (SNPs) compared with MZ977164, constituting a new sequence type. The *C. bovis* XXVIb sequence is identical to MZ977195, whereas the XXVIe sequence constitutes a new variant. The *C. bovis* subtypes XXVIe and XXVIf were the dominant subtypes in animals in this study. As expected, subtype families of *C. bovis* clustered with these reference sequences in phylogenetic analysis of the *gp60* gene (Figure 2b). Of the 65 *C. ryanae*-positive samples, 46 were successfully subtyped, revealing seven subtype families: XXIa (n = 6), XXIb (n = 5), XXIc (n = 1), XXIf (n = 5), XXIi (n = 2), XXIj (n = 24), and XXIk (n = 3). Among those identified in this study, *C. ryanae* subtype XXIj was the most common (Table 1). The nucleotide sequence of *C. ryanae* XXIi represented a novel variant as it differed from OP925778 by one SNP. *Cryptosporidium ryanae* XXIj was also a novel variant as it presented 3 SNPs and an 18 nt insertion, and XXIk was indeed identical to OP925782. The sequences from the subtypes XXIa and XXIb differed from OP925788 and OP925776 by three and two SNPs, respectively. The sequences of subtype XXIc exhibited 99.6% nucleotide identity with OP925777. The sequences of subtype XXIf represented a new sequence type as they contained several SNPs and were shorter than previously reported sequences. As expected, the subtype families of *C. ryanae* clustered with their corresponding reference sequences in the phylogenetic analysis of the *gp60* gene (Figure 2c).

## 4. Discussion

The findings of this study reveal that *Cryptosporidium* spp. are prevalent in dairy cattle from Fuyang, Huainan, Huaibei, and Bengbu in Anhui Province, China. The overall *Cryptosporidium* positivity rate was 35.7% (338/948), which is consistent with a report from Taiwan (37.6%, 173/460) [25]. In contrast, the positivity rate was significantly higher than that in neighbouring provinces: Jiangsu (19.1%, 251/1315) [26], Shandong (24.3%, 36/148) [27], and Henan (16.7%, 727/4348) [28]. In Anhui Province, *Cryptosporidium* infections displayed pronounced spatial heterogeneity and age-associated trends. Positivity rates differed significantly across the four cities, with Bengbu exhibiting the highest rate (61.5%; 190/309). This geographical variation is likely driven by regional differences in farm management practices and ecological factors. For instance, Fuyang, a newly established farming area, may have introduced healthier cattle with no prior infection history, resulting in a lower initial positivity rate. In contrast, Bengbu, a traditional high-density farming region, likely exacerbated pathogen transmission due to intensive practices [29]. Longer-established farms tend to have higher environmental oocyst contamination loads, thereby increasing the risk of infection in animals [2,11,21].

*Cryptosporidium* age-specific prevalence aligns with global patterns, exhibiting the highest positivity in young animals in farm-level analysis, particularly calves < 2 months old [29]. For example, Huainan calves < 2 months exhibited a 34.3% (12/35) positivity rate. *Cryptosporidium parvum* infections decreased with age, while *C. andersoni* became dominant, consistent with findings in Henan [30]. *Cryptosporidium parvum* primarily infects calves < 2 months, and *C. andersoni* prevails in adult cattle [31,32,33]. In Anhui dairy cattle, the highest prevalence was observed in the 2–6 months age group (43.1%, 155/359), whereas calves < 2 months had a lower prevalence (27.2%, 88/323). The apparent differences between the provincial- and farm-level models are likely attributable to the relatively small sample sizes in the analyses.

*Cryptosporidium parvum* subtype IIdA20G1 was detected in Huainan and Huaibei, whereas subtype IIdA15G1 was only recorded in Bengbu, suggesting a geographical influence on genetic diversity. These subtypes belong to the IId family, which also includes subtypes IIdA19G1 and IIdA15G1 in Gansu [34], IIdA19G1 in Henan [35], and IIdA15G1 in Ningxia [36]. The IId subtype family predominates in Chinese cattle populations and exhibits zoonotic potential [14,35]. The high prevalence of IId subtypes in Anhui highlights the potential risk of dairy cattle as zoonotic reservoirs. In the present study, five *C. bovis* subtype families (XXVIa, XXVIb, XXVId, XXVIe, and XXVIf) were identified, whereas subtype family XXVIc was also detected in previous studies [37]. The dominant subtype family was XXVIe (39/99) in this study, which is consistent with reports from Guangdong in two different studies [18,37]. In this study, XXVIf was also the predominant subtype family in dairy cattle; however, in one study, researchers identified the XXVIf subtype family as the predominant subtype in yaks [18]. This finding was derived from a limited sample (n = 26), which may limit representativeness. *Cryptosporidium bovis* subtypes presented a broader geographical range, with subtypes XXVIb and XXVIe identified in three of the four farms and XXVIa, XXVId, and XXVIf in two. The subtype families of *C. ryanae* were first described, and a total of eight subtype families (XXIa-XXIh) were identified [17]. In a recent study, the authors reported three additional subtype families (XXIi-XXIk) of *C. ryanae* [37]. In this study, seven subtype families (XXIa, XXIb, XXIc, XXIf, XXIi, XXIj, and XXIk) were identified. The dominant subtype family identified is XXIj, not XXIa, as previously reported in dairy cattle [17], in agreement with a report from a farm in Guangdong [37] and contrary to the subtype family XXIa, as previously reported in dairy cattle. Subtype distribution of *C. ryanae* differed geographically, with XXIa, XXIf, and XXIj detected exclusively in Bengbu; XXIc and XXIk only in Huaibei; XXIb in both Huainan and Huaibei; and XXIi in Fuyang and Huaibei.

These findings demonstrate significant geographical specificity and age dependency in *Cryptosporidium* transmission among Anhui dairy cattle, providing critical insights for controlling *Cryptosporidium* spp. in eastern China. The persistently high prevalence in hotspot regions such as Bengbu and Huaibei underscores the need for targeted control strategies tailored to regional epidemiological patterns. Priority should be given to enhanced protection for calves < 6 months old, combined with strengthened biosecurity management in areas with co-circulating pathogens. This study was limited to four farms in Anhui Province, and the findings may not be generalisable to other regions of China.

## 5. Conclusions

In conclusion, the results of the study have demonstrated a common occurrence of *Cryptosporidium* species and subtypes in dairy cattle in Anhui, China. Four *Cryptosporidium* species were identified, namely, *C. parvum*, *C. andersoni*, *C. bovis*, and *C. ryanae*. Subtype analysis revealed that *C. parvum* isolates belonged to the IId subtype families (IIdA15G1 and IIdA20G1), while five subtype families of *C. bovis* and seven subtype families of *C. ryanae* were identified, with distinct geographical distributions. The identification of zoonotic *C. parvum* IId subtypes highlights the potential public health risk associated with dairy cattle in Anhui. Notably, in this study, we provide the first subtype analysis of *C. bovis* and *C. ryanae* in dairy cattle in Anhui, offering new insights into the genetic diversity of these two species in this region. These findings provide a scientific basis for developing region-specific control strategies.

## Figures and Tables

**Table 1:** Distribution of *Cryptosporidium* spp. in cattle on farms in Anhui Province, China.

Location	Age (Months)	No. Specimens	No. Positive (%)	*Cryptosporidium* spp.				*C. parvum* Subtype	*C. ryanae* Subtype	*C. bovis* Subtype
				*C. parvum*	*C. ryanae*	*C. bovis*	*C. andersoni*			
Fuyang	<2	215	20 (9.3)	-	2	18	-	-	XXIi (1)	XXVIb (5), XXVId (7), XXVIf (3)
	2–6	19	0 (0.0)	-	-	-	-	-	-	-
	subtotal	234	20 (8.5)	-	2	18	-	-	XXIi (1)	XXVIb (5), XXVId (7), XXVIf (3)
Huainan	<2	35	12 (34.3)	12	-	-	-	IIdA20G1 (12)	-	-
	2–6	43	14 (32.6)	11	1	1	1	IIdA20G1 (9)	XXIb (1)	XXVIe (1)
	6–12	69	2 (2.9)	-	1	1	-	-	-	-
	>12	20	5 (25.0)	-	-	-	5	-	-	-
	subtotal	167	33 (19.8)	23	2	2	6	IIdA20G1 (21)	XXIb (1)	XXVIe (1)
Huaibei	<2	19	15 (78.9)	-	3	12	-	-	XXIb (1)	XXVIe (8)
	2–6	153	58 (37.9)	46	8	4	-	IIdA20G1 (39)	XXIb (3), XXIc (1), XXIk (3)	XXVIe (2)
	6–12	28	10 (35.7)	5	1	4	-	IIdA20G1 (3)	XXIi (1)	XXVIa (1), XXVIb (1), XXVId (2)
	>12	38	12 (18.2)	4	2	2	4	IIdA20G1 (1)	-	-
	subtotal	238	95 (39.9)	55	14	22	4	IIdA20G1 (43)	XXIb (4), XXIc (1), XXIi (1), XXIk (3)	XXVIa (1), XXVIb (1), XXVId (2), XXVIe (10)
Bengbu	<2	54	41(75.9)	20	6	12	3	IIdA15G1 (16)	XXIj (2)	XXVIf (8)
	2–6	144	83 (57.6)	16	31	34	2	IIdA15G1 (11)	XXIa (6), XXIf (5), XXIj (17)	XXVIe (28), XXVIf (3)
	6–12	58	36 (62.1)	3	5	27	1	IIdA15G1 (2)	XXIj (2)	XXVIa (4), XXVIb (3), XXVIf (17)
	>12	53	30 (56.6)	7	5	10	8	IIdA15G1 (5)	XXIj (3)	XXVIf (6)
	subtotal	309	190 (61.5)	46	47	83	14	IIdA15G1 (34)	XXIa (6), XXIf (5), XXIj (24)	XXVIa (4), XXVIb (3), XXVIe (28), XXVIf (34)
Total		948	338 (35.7)	124	65	125	24	IIdA15G1 (34), IIdA20G1 (64)	XXIa (6), XXIb (5), XXIc (1), XXIf (5), XXIi (2), XXIj (24), XXIk (3)	XXVIa (5), XXVIb (9), XXVId (9), XXVIe (39), XXVIf (37)

**Table 2:** Occurrence of *Cryptosporidium* spp. in cattle on farms in Anhui Province, China, stratified by age.

Age (Months)	No. Specimens	No. Positive (%)	*Cryptosporidium* spp.				*C. parvum* Subtype	*C. ryanae* Subtype	*C. bovis* Subtype
			*C. parvum*	*C. ryanae*	*C. bovis*	*C. andersoni*			
<2	323	88 (27.2)	32	11	42	3	IIdA15G1 (16), IIdA20G1 (12)	XXIi (1), XXIb (1), XXIj (2)	XXVIb (5), XXVId (7), XXVIe (8), XXVIf (11)
2–6	359	155 (43.1)	73	40	39	3	IIdA15G1 (11), IIdA20G1 (48)	XXIa (6), XXIb (4), XXIc (1), XXIf (5), XXIj (17), XXIk (3)	XXVIe (31), XXVIf (3)
6–12	155	48 (30.9)	8	7	32	1	IIdA15G1 (2), IIdA20G1 (3)	XXIi (1), XXIj (2)	XXVIa (5), XXVIb (4), XXVId (2) XXVIf (17)
>12	111	47 (42.3)	11	7	12	17	IIdA15G1 (5), IIdA20G1 (1)	XXIj (3)	XXIf (6)
Total	948	338 (35.7)	124	65	125	24	IIdA15G1 (34), IIdA20G1 (64)	XXIa (6), XXIb (5), XXIc (1), XXIf (5), XXIi (2), XXIj (24), XXIk (3)	XXVIa (5), XXVIb (9), XXVId (9), XXVIe (39), XXVIf (37)

## Data Availability

Representative nucleotide sequences generated were submitted to the GenBank database under accession the numbers PZ496286-PZ496292 and PZ786721-PZ786727 in this study.
